# Improving the Topside Profile of Ionosonde with TEC Retrieved from Spaceborne Polarimetric SAR

**DOI:** 10.3390/s19030516

**Published:** 2019-01-26

**Authors:** Cheng Wang, Wulong Guo, Haisheng Zhao, Liang Chen, Yiwen Wei, Yuanyuan Zhang

**Affiliations:** 1 Qian Xuesen Laboratory of Space Technology, China Academy of Space Technology, Haidian district, Beijing 100094, China; guo.wulong@163.com (W.G.); chenliang@qxslab.cn (L.C.); 2 National Key Laboratory of Electromagnetic Environment, China Research Institute of Radiowave Propagation, Qingdao 266107, China; zhaohaisheng213@163.com; 3 School of Physics and Optoelectronic Engineering, Xidian University, Xi’an 710071, China; ywwei@xidian.edu.cn (Y.W.); yyzhang1@xidian.edu.cn (Y.Z.)

**Keywords:** polarimetric synthetic aperture radar, total electron content, ionospheric electron density distribution

## Abstract

Signals from spaceborne polarimetric synthetic aperture radar will suffer from Faraday rotations when they propagate through the ionosphere, especially those at L-band or lower frequencies, such as signals from the Phased Array type L-band Synthetic Aperture Radar (PALSAR). For this reason, Faraday rotation compensation should be considered. On the other hand, Faraday rotation could also be retrieved from distorted echoes. Moreover, combining Faraday rotation with the radar parameters and the model of magnetic field, we could derive the total electron content (TEC) along the signal path. Benefiting from the high spatial resolution of the SAR system, TEC obtained from PALSAR could be orders of magnitude higher in spatial resolution than that from GPS. Besides, we demonstrated that the precision of TEC from PALSAR is also much higher than that from GPS. With the precise TEC available, we could fuse it with data from other ionosphere detection devices to improve their performances. In this paper, we adopted it to help modify the empirically modeled topside profile of ionosonde. The results show that the divergence between the modified profile and the referenced incoherent scattering radar profile reduced by about 30 percent when compared to the original ionosonde topside profile.

## 1. Introduction

Ionospheric variations have been studied for earthquake prediction, solar activity analysis, radar image modification and geomagnetic storm research [1,2,3,4,5]. Among all kinds of ionospheric characteristics, the total electron content (TEC) is one of the most used parameters. For TEC evaluation, widely used instruments include Incoherent Scatter Radar (ISR), ionosonde, and Global Navigation Satellite Systems (GNSS). Beyond their popularity, some inconveniences still exist for each instrument. The ISR is the most powerful piece of equipment for TEC detection. However, it is poorly distributed on the Earth due to its expensive cost for developing and operation. The ionosonde station is easy to build and has a reasonable cost, so it has been set up worldwide [6]. Nevertheless, an inherent issue of the ionosonde is that it can only directly detect the electron density under the peak height. Though many methods have been proposed for modeling the topside profile of ionosphere from the ionosonde measurement, it is still a subject for ongoing investigation [7,8,9]. The GNSS can map the TEC of the ionosphere globally and in real-time, but the spatial resolution of the observation is too low for the fine analysis of ionosphere above a certain area [10]. Recently, full polarimetric spaceborne synthetic aperture radar (PolSAR) was demonstrated to be qualified for ionospheric inhomogeneities imaging [11]. Specifically, Faraday rotation (FR) and TEC images were derived from PolSAR data, and ionospheric perturbations observed from variations of these images were verified using ground-based GPS receivers and network. Instead of analyzing relative variations in TEC images, we would like to quantitatively determine the precision of TEC retrieved from PolSAR, i.e., how precise the TEC could be when compared to that obtained from the powerful ISR. Furthermore, we would like to present a method for improving the topside electron density profile of ionosonde using the precise TEC retrieved from PolSAR. 

This work relates to two parts of ionosphere detection. First, we evaluated the precision of TEC measured by the spaceborne synthetic aperture radar. Experiments showed impressive results whereby the precision was within 1 TECU when compared with the TEC obtained from ISR. Specifically, as examples, we used the L band PolSAR data from the Phased Array type L-band Synthetic Aperture Radar (PALSAR) onboard the Advanced Land Observation Satellite. The ISR station that observes the same space as the PolSAR was found. Then, TECs derived from these two facilities were compared. The results verified their consistency. 

The second part of this paper presents a method to improve the topside profile of the ionosonde with TEC retrieved from PolSAR data. The ionosonde station located in the scene of PolSAR was found. Since the ionosonde could directly observe the electron densities under the peak height, we were then able to derive the TEC of the topside profile by subtracting the TEC of the bottomside profile from the PolSAR TEC. This topside TEC could then be used to help model the electron density topside profile. To evaluate our method, we managed to find an ISR which was also located in the same area as the ionosonde station. A comparison of the electron density profile of the ISR and the modified topside profile of ionosonde validated our algorithm. 

The outline of this paper is given as follows: Section 2 presents the method of TEC retrieval from PolSAR and the method for improving the topside profile model of ionosonde with the known TEC. Then, details of data used in this paper are introduced in Section 3. Section 4 illustrates our experimental results to validate our proposed methods. Finally, Section 5 concludes the paper.

## 2. Methods for TEC Retrieval and Topside Profile Modification

We first present the methods used in this paper for retrieving TEC from PolSAR data, and then introduce the algorithm for improving the topside profile of ionosonde with the retrieved TEC.

### 2.1. TEC Retrieval from PolSAR

Radar signals at the L band and lower frequencies will suffer strong Faraday rotation (FR) effects when they pass the ionosphere. However, these FR effects could serve as useful information for deriving the total electron density along the signal propagation path. According to [11,12], the TEC along the radio path of PolSAR is related to FR by

(1) $$\Omega =\frac{2.365\times 10^{4}}{f^{2}}\int n_{e}B\cos\theta ds=\frac{2.365\times 10^{4}}{f^{2}}\langle B\cos\theta \rangle TEC,$$

where Ω stands for FR, f is the radar operating frequency, B denotes the magnitude of the ambient magnetic field, and θ is the angle between the radio wave and ambient magnetic field vectors. The second equality in Equation (1) is deduced from the first mean value theorem for definite integrals [13], where $TEC=\int n_{e}ds$ is the TEC along the radar signal path, and 〈Bcosθ〉 corresponds to the mid-value of Bcosθ at a specific altitude of the path. Therefore, once FR is estimated from distorted radar signals, we can easily derive TEC under a specified 〈Bcosθ〉.

FR retrieval has been well studied in the last two decades [14,15,16,17,18,19,20,21]. In this paper we chose the B&B method [16] as the FR estimator due to its robustness and reliability:

(2) $$\begin{array}{c} \left[\begin{array}{cc} Z_{11} & Z_{12}\\ Z_{21} & Z_{22} \end{array}\right]=\left[\begin{array}{cc} 1 & j\\ j & 1 \end{array}\right]\left[\begin{array}{cc} M_{hh} & M_{vh}\\ M_{vh} & M_{vv} \end{array}\right]\left[\begin{array}{cc} 1 & j\\ j & 1 \end{array}\right]\\ \Omega =\frac{1}{4}\arg\left(\langle Z_{12}Z_{21}^{*}\rangle \right). \end{array}$$

In Equation (2), the M matrix represents the measured full polarimetric scattering matrix. Therefore, when the full polarimetric data is obtained, TEC can be calculated from Equations (1) and (2).

### 2.2. Improving the Topside Profile Model of Ionosonde with Known TEC

Many models are available for the topside profile of ionosonde. In this paper, we aim to improve the most commonly used model, the α-Chapman model, with a constant scale height $H_{T}$ [7]:

(3) $$\begin{array}{c} Ne=N_{m}F_{2}\times e^{\frac{1-z-e^{-z}}{2}}\\ z=\frac{h-h_{m}F_{2}}{H_{T}} \end{array},$$

where $N_{m}F_{2}$ represents the electron density at the peak height $h_{m}F_{2}$ of the $F_{2}$ layer. Both $N_{m}F_{2}$ and $h_{m}F_{2}$ can be directly obtained from the ionosonde station. Therefore, $H_{T}$ is the only parameter that we need to estimate to determine a topside profile. 

Traditional method estimates $H_{T}$ from the bottomside profile of each measurement [9]. Though empirically feasible, this method does not take into account any truly measured characteristic about the topside profile. In this paper, we propose a method to evaluate $H_{T}$ with the TEC of the topside density profile. Integrating Equation (3) with respect to height gives

(4) $$\frac{1}{{\left(N_{m}F_{2}\right)}^{2}}\int Ne^{2}dh=\int e^{1-z-e^{-z}}dh.$$

According to [22,23,24], if the electron density profile is Chapman-model based, we have

(5) $$\int Ne^{2}dh=0.66Ne_{\max}TEC,$$

where $Ne_{\max}$ is the maximal electron density along the path. Referring to the topside profile only, we can obtain from Equation (5)

(6) $$\int_{h_{m}F_{2}}^{H_{S}}Ne^{2}dh=0.66Ne_{\max}TEC_{Top}.$$

$TEC_{Top}$ is the TEC of the topside profile of the ionosonde. When we can measure the TEC through other equipment precisely, $TEC_{Top}$ is simply obtained by subtracting $TEC_{Bott}=\int_{0}^{h_{m}F_{2}}Nedh$ from TEC. Substituting Equation (6) into the left side of Equation (4) gives

(7) $$\frac{0.66Ne_{\max}TEC_{Top}}{{\left(N_{m}F_{2}\right)}^{2}}=\int_{h_{m}F_{2}}^{H_{s}}e^{1-z-e^{-z}}dh.$$

In Equation (7), $H_{s}$ is the maximum altitude to which the TEC is measured, and in this paper, it is the altitude of PALSAR. Integrating the right side of Equation (7) while recalling Equation (3), we can obtain

(8) $$\int_{h_{m}F_{2}}^{H_{s}}e^{1-z-e^{-z}}dh=H_{T}\int_{0}^{\frac{H_{s}-h_{m}F_{2}}{H_{T}}}e^{1-z-e^{-z}}dz=H_{T}\left(\exp\left(1-e^{-\frac{H_{s}-h_{m}F_{2}}{H_{T}}}\right)-1\right).$$

Joining Equations (7)–(8) leads to

(9) $$\frac{0.66Ne_{\max}TEC_{Top}}{{\left(N_{m}F_{2}\right)}^{2}}=H_{T}\left(\exp\left(1-e^{-\frac{H_{s}-h_{m}F_{2}}{H_{T}}}\right)-1\right)$$

Solving the nonlinear Equation (9), we can obtain $H_{T}$. Thus, the topside profile can be obtained from Equation (3) with the TEC-related constant scale height $H_{T}$.

## 3. Data Details

### 3.1. Data for Estimating TEC Precision of PolSAR

**Figure 1:**
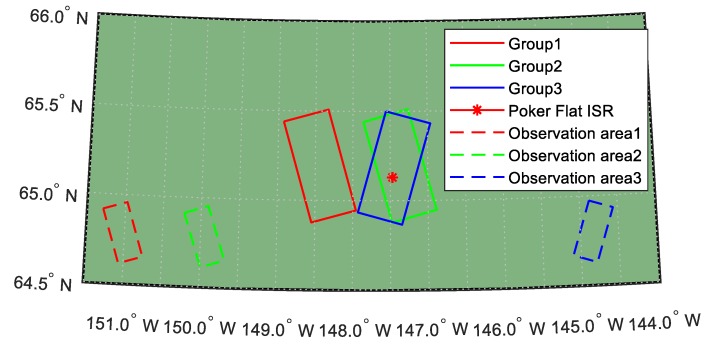
Position relationship of three ground tracks of PALSAR scenes and Poker Flat ISR. The red rectangle corresponds to the PALSAR scene of Group 1, while the green and the blue ones correspond to the measurement of Group 2 and Group 3, respectively. The position of Poker Flat ISR is shown as a red asterisk, while the observation area of each group is shown as a dashed rectangle in its corresponding color.

The spaceborne L band PolSAR data for TEC retrieval were obtained from the Phased Array type L-band Synthetic Aperture Radar (PALSAR) of the Japanese Advanced Land Observation Satellite (ALOS) which orbits Sun-synchronously at about 692 km of altitude (data access https://vertex.daac.asf.alaska.edu/). Each scene of the radar data covers a rectangle area of the Earth. The TEC of each scene is estimated as the average TEC of the whole scene. For a quantitative evaluation of the reliability of TEC derived from PolSAR, we compared it to the TEC derived from ISR, because ISR is believed to be the most powerful device for monitoring the ionosphere. However, since ISRs are sparsely distributed on the earth, despite trying our best, we only managed to find three groups of corresponding data. The ISR data were collected from Poker Flat ISR station [25], and corresponding PolSAR scenes were chosen to make sure the distance from the Poker Flat ISR to the scene was less than 40 km. Three selected scenes are shown in Figure 1.

From Figure 1, we can find that Poker Flat ISR station is fully covered by PALSAR scenes of Group 2 and 3, while the range between the ISR station and PALSAR scene 1 is within 40 km. The PALSAR illumination modes of three scenes can also be observed in Figure 1. Specifically, the dashed rectangles represent the actual observation areas of PALSAR on ionosphere at 300 km, i.e., the coverage of PALSAR beam on the ionosphere at 300 km. Therefore, we know that the distance from each observation area to its ground scene center is around 100 km and that the TEC obtained from PALSAR is slant TEC rather than vertical TEC (VTEC). The VETC should be estimated as

(10) VTEC=TEC×cosη,

where η is the off-nadir angle of the PALSAR. To ensure that the ISR and PALSAR observe the same space, the best choice is to find an observation of ISR that just to illuminate the area of PALSAR. However, this kind of observation is not available, so we picked the observation closest to that of PALSAR as a representation and assumed that the ionosphere is stable within the observation area. Details about the observations are given in Table 1. The Piercing Lat. (Lon.) in the table is the center latitude (longitude) of the observation area of the corresponding instrument. From the Table 1, we can see that the two devices observed the same ionosphere at the same time.

### 3.2. Data for Modeling the Topside Profile of Ionosonde

Once the TEC of an area was calculated from PolSAR data, it was possible to utilize it to improve the topside profile of the ionosonde that observes the same area with PolSAR. In this paper, the ionosonde data was accessed from Digital Ionogram DataBase (DIDB) [26]. The topside profile of the data was based on what we discussed previously, i.e., the α-Chapman model with a constant scale height $H_{T}$. Therefore, a comparison of the original profile and the modified profile is available. As a reference of the true measured electron profile, ISR data was again adopted in the experiment to validate our proposed method. 

**Figure 2:**
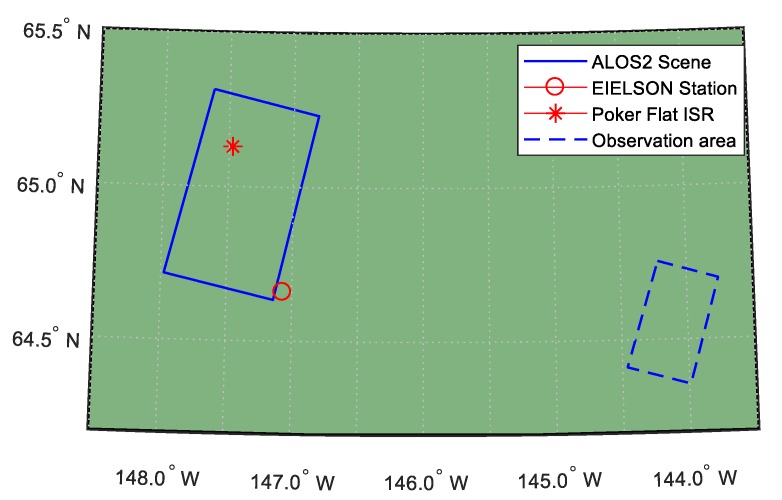
Geographic relationship of ALOS-2 scene, EIELSON station, and Poker ISR station.

Despite doing our best, we only found one group of corresponding data, as shown in Table 2. The ISR data was still obtained from Poker Flat ISR station, the ionosonde data was from EIELSON station (ID: EI764), and the PolSAR data was from PALSAR-2 on board Advanced Land Observing Satellite-2 (ALOS-2). Their geographic relationship is shown in Figure 2 where the PolSAR scene fully covers the Poker ISR station and EIELSON station is adjacent to the right bottom of the scene. From the “Piercing Lat.” and “Piercing Lon.” in Table 2, we could know that both ISR and ionosonde observe the ionosphere vertically. Therefore, we only need to pay attention to the off-nadir angel of PALSAR while estimating the VTEC. 

## 4. Results and Discussions

### 4.1. Validation of the Precision of PolSAR in Estimating TEC

Integrating the electron density profile of ISR up to the altitude of PALSAR gave the TEC of ISR along the observing direction. The VTEC of ISR was obtained by using Equation (10), but the η of ISR is the angle between the beam direction and the vertical direction.

Equation (1) tells us that the value of PolSAR TEC is affected by the mid-value 〈Bcosθ〉 [13]. However, it is not easy to theoretically determine such a value. In our experience, the value of Bcosθ linearly decreases as the altitude increases, so the mid-value is determined mostly by the electron density profile. Here, we give a brief discussion about how to determine the 〈Bcosθ〉. Recall Equation (1) and explicitly represent it into the form of the first mean value theorem for definite integrals:

(11) $$\Omega =\frac{2.365\times 10^{4}}{f^{2}}\int n_{e}B\cos\theta ds=\frac{2.365\times 10^{4}}{f^{2}}\int_{0}^{H_{s}}B\cos\theta dTEC=\frac{2.365\times 10^{4}}{f^{2}}\langle B\cos\theta \rangle TEC.$$

Comparing Equation (11) to the first mean value theorem for definite integrals, we get

(12) $$\int_{a}^{b}f\left(x\right)dx=f\left(\xi \right)\left(b-a\right),\xi \in \left[a,b\right]$$

It can be found that Bcosθ corresponds to f(x) and TEC corresponds to x. Therefore, to determine the mid-value 〈Bcosθ〉, we should know the functional relationship between Bcosθ and TEC. Though the function cannot be explicitly represented, we can show it numerically in a figure and obtain some useful information. 

**Figure 3:**
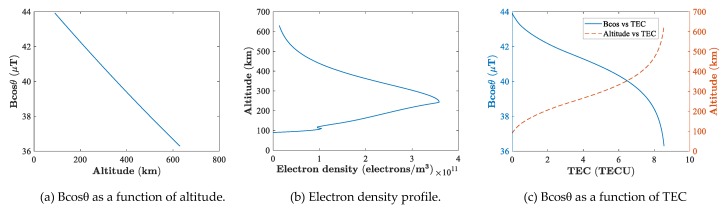
Relationship between altitude and total electron content (TEC) as well as Bcosθ.

Take the data from ionosonde and PolSAR in Table 2 as an example. The Bcosθ as a function of altitude could be obtained from the ambient magnetic field of PALSAR, as shown in Figure 3a. Clearly in Figure 3a,Bcosθ decreases nearly linearly with the increase of altitude. TEC as a function of altitude could be obtained by integrating the electron density profile of the ionosonde in Figure 3b. Taking advantage of the same variable, i.e., the altitude in the above two functions, we can plot Bcosθ as a function of TEC, as shown in Figure 3c. For clarity, the relationship between altitude and TEC is plotted in Figure 3c, so we can get a direct idea about the altitude where the mid-value 〈Bcosθ〉 lies. Bcosθ still monotonically decreases in Figure 3c, but the curve changes from convex to concave at the peak height. 

**Figure 4:**
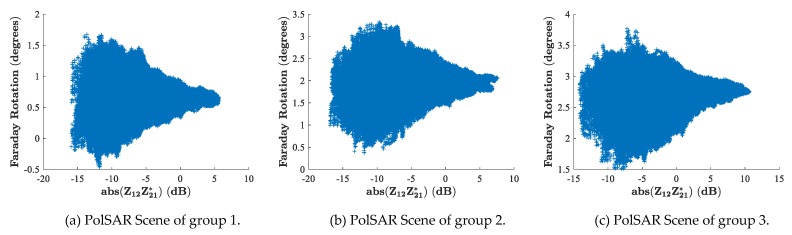
Faraday rotation (FR) estimates as a function of signal amplitude. PolSAR: polarimetric spaceborne synthetic aperture radar.

The first mean value theorem for definite integrals shows that 〈Bcosθ〉TEC is actually the area of the region under the “Bcosθ vs. TEC” curve of Figure 3c. Intuitively, the mid-value 〈Bcosθ〉 of Figure 3c should be located at around 5 TECU which corresponds to an altitude of about 400 km. Though we only take one group of data as an example, most cases satisfy this mode where the altitude for the mid-value 〈Bcosθ〉 is a little higher than the peak height of the ionosphere as long as the TEC of the topside profile is larger than the TEC of the bottom side. However, it was still not possible for us to determine a specific mid-value for each scene of PolSAR, so we experimented on three different altitudes to see how precisely the PolSAR could estimate TEC and how the magnetic field influences the TEC value. The magnetic field at 300 km, 400 km, and 500 km was picked as the mid-value along each radio path, respectively. For each scene of PolSAR, FR estimation from Equation (2) may be biased by residual calibration errors, though PALSAR has been reported to be well calibrated [27,28]. Therefore, following [29], only pixels of SNR >10 dB were selected to estimate the final TEC. This corresponds to select pixels of a signal amplitude higher than −17 dB, since the noise equivalent sigma zero (NESZ) is estimated to be about −27 dB for PALSAR. Here, FR estimates as a function of the signal amplitude are given in Figure 4 for each PolSAR scene, where the signal amplitude is defined as the “circular cross-pol product” $\mathrm{abs}\left(Z_{12}Z_{12}^{*}\right)$ from Equation (2). From Figure 4, we can see that the signal amplitude of each PolSAR scene is higher than −17 dB. Therefore, the TEC derived from averaging the whole PolSAR TEC image is countable. One should note that the average window used to reduce the speckle noise in Equation (2) was set to be 21×41 pixels. Actually, we tested window sizes from 10×10 to 200×200, and the changes between the resulting TECs were within 0.01 TECU.

Results of TECs retrieved from different instruments are given in Table 3. The TEC deviation at 400 km is defined as the absolute value of the difference between PALSAR VTEC and ISR VTEC. Since TEC varies in a PolSAR scene, we calculated the standard deviation of TEC for each PolSAR scene and present it following each term in parentheses. All figures in Table 3 are given in units of TECU (1 TECU = 10^16^ electrons/m^2^).

From Table 3, some phenomena are observed. First, the standard deviation of TEC in each PolSAR scene is small. This feature demonstrates that no strong variation occurred in the ionosphere during the observations. Therefore, the average TEC of the scene can be represented by the PolSAR TEC. Second, the derived PolSAR TEC increases along with the altitude of the magnetic field, which can be easily explained from Equation (1). Since 〈Bcosθ〉 decreases with an increase in altitude, the derived TEC will get larger. Third, the TEC deviations between ISR and PolSAR are within 1 TECU. This illustrates the feasibility of measuring TEC with PolSAR. For a better understanding of the performance of PolSAR, we also present here the TECs obtained from GNSS. Clearly, the TEC differences between GNSS and ISR range from 3 to 5 TECU. Since GNSS measures TEC up to about 20,000 km, the differences are acceptable. However, for group 1 and group 2, the TECs from GNSS are way too large to be believed as precise. For a further illustration that the ionosphere is stable during the measuring time, we also give the average TEC of ISR in different directions. The ISR data belonging to different beam directions were first collected together, and a polynomial fitting algorithm was adopted to form a smooth electron density profile. Then average TEC of ISR was obtained by integrating the electron densities with respect to the altitude. The small differences between “Poker Flat ISR” and “Poker Flat ISR (average)” further verify the stable circumstance of the ionosphere.

### 4.2. Modeling the Topside Profile of Ionosonde with New H_T_

We have demonstrated that PolSAR data can be used to estimate the TEC under 700 km with a considerable precision within 1 TECU. Therefore, after the bottomside electron density profile of ionosphere has been precisely measured by an ionosonde, the corresponding PolSAR TEC can be used to calculate the *TEC_Top_* for the ionosonde. Resorting to Equation (9), we can derive the parameter $H_{T}$. Then, the topside profile could be easily calculated from Equation (3). In this section, PolSAR TEC derived with magnetic field at 400 km is used, which is 11.4 TECU. Still, to account for potential residual calibration errors, only pixels of SNR > 10 dB are employed. For clarity, the TECs of “Poker Flat ISR” and “Poker Flat ISR (average)” are also given here, which are 12.37 TECU and 12.23 TECU, respectively. Clearly, the deviation between PolSAR TEC and ISR TEC is still within 1 TECU, which again validates the precision of PolSAR for TEC estimation. The small difference between “Poker Flat ISR” and “Poker Flat ISR (average)” indicates a stable ionosphere. For comparison, the TEC obtained from GNSS is 15.7 TECU, which is acceptable but not as precise as that of PolSAR.

**Figure 5:**
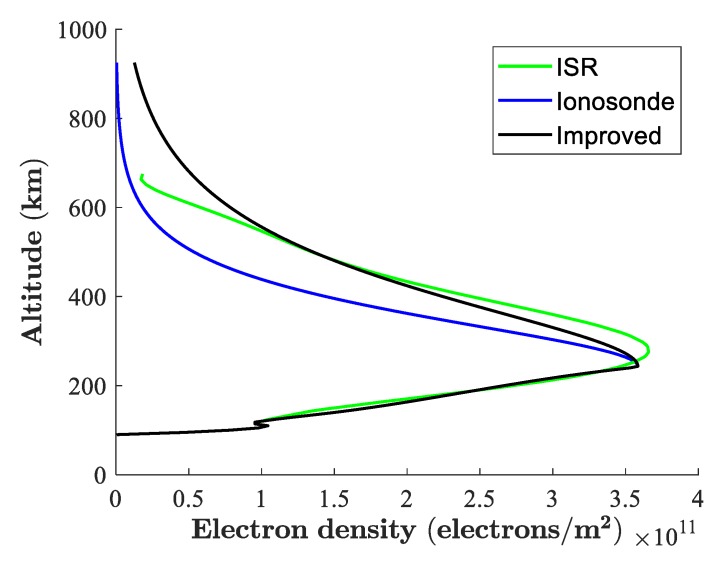
Electron density profiles for ISR and ionosonde.

Figure 5 shows the result of the proposed method where “ISR” represents the ISR data after polynomial fitting. Note that we adopted the ISR data from all beams for polynomial fitting, rather than the vertical beam only, because the TEC deviations between “Poker Flat ISR” and “Poker Flat ISR (average)” are small, and vertical data is too sparse to form a fine polynomial fitting. In Figure 5, “Ionosonde” stands for the original data obtained from DIDB, and “Improved” is our result with the topside profile calculated from the known *TEC_Top_*. It is clear that the bottomside profile of ionosonde matches well with that of ISR, while the two topside parts are divergent. The reason for this was discussed previously, i.e., that no topside information is considered in the original method. After modifying the topside profile with the known TEC, we can easily see that the new topside profile is more consistent with that of ISR. The divergence between the ionosonde topside profile and the ISR topside profile reduced by 30.41 percent after the presented algorithm was adopted. Here, the divergence is defined as the average absolute difference between the ISR profile and the ionosonde profile, and the percentage of reduction was calculated from Equation (13). This result proves the validity of our method.

(13) $$percentage=1-mean\left(\frac{{\left|ISR-Improved\right|}_{Top}}{{\left|ISR-Ionosonde\right|}_{Top}}\right).$$

## 5. Conclusions

This paper first validated the ability of PolSAR as an effective device in measuring the TEC of the ionosphere, and then demonstrated the feasibility of using known TEC to help improve the topside profile of ionosonde. The results show that PolSAR is able to measure the TEC with higher precision compared to GNSS. Furthermore, the improved topside profile proved to be much more consistent with the profile of ISR than the original profile. 

In this paper, the α-Chapman model was used for the topside profile because of its popularity. Actually, if any other model is developed for the topside profile, the proposed TEC modification strategy could also be taken as an auxiliary process. One should note that the proposed method could only detect the TEC under the altitude of the satellite. However, it is still reliable to expand the modified ionosonde profile beyond this altitude, since TECs under 700 km cover the majority of TECs under 10,000 km.

## Figures and Tables

**Table 1:** Corresponding observing times and positions of three groups of data. The off-nadir angle of PALSAR (Phased Array type L-band Synthetic Aperture Radar) in this table is 21.5 degrees. ISR: Incoherent Scatter Radar.

Data Group	Instrument	Center Latitude	Center Longitude	Center Observation Time (UTC:Y/M/D HH/MM)	Piercing Latitude	Piercing Lontitude
1	PALSAR	65.193	−148.439	2011/03/19 07/32	64.786	−151.022
	Poker Flat ISR	65.130	−147.471	2011/03/19 07/30	64.650	−148.000
2	PALSAR	65.194	−147.369	2011/03/31 07/28	64.782	−149.949
	Poker Flat ISR	65.130	−147.471	2011/03/31 07/28	65.130	−147.471
3	PALSAR	65.183	−147.450	2010/08/06 21/06	64.800	−144.832
	Poker Flat ISR	65.130	−147.471	2010/08/06 21/08	65.370	−145.070

**Table 2:** Observing time and positions of three instruments. The off-nadir angle of PALSAR-2 in this table is 30.8 degree.

Instrument	Center Latitude	Center Longitude	Center Observation Time (UTC: Y/M/D HH/MM)	Piercing Latitude	Piercing Longitude
PALSAR	65.193	−148.439	2014/08/29 22/24	64.603	−144.362
Poker Flat ISR	65.130	−147.471	2014/08/29 22/20	65.130	−147.471
EIELSON station	64.660	−147.070	2014/08/29 22/15	64.660	−147.070

**Table 3:** TEC measurements of Poker Flat ISR and PolSAR. GNSS: Global Navigation Satellite System.

Instrument.	Group 1	Group 2	Group 3
Poker Flat ISR	1.484	5.700	7.365
PolSAR (300 km)	1.514 (0.324)	4.646 (0.444)	6.812 (0.351)
PolSAR (400 km)	1.586 (0.340)	4.864 (0.465)	7.112 (0.367)
PolSAR (500 km)	1.660 (0.355)	5.092 (0.487)	7.434 (0.384)
Deviation (400 km)	0.102	0.836	0.253
Poker Flat ISR (average)	1.46	5.71	7.86
GNSS	6	12.5	10.7
